# Terahertz Time-of-Flight Ranging with Adaptive Clock Asynchronous Optical Sampling

**DOI:** 10.3390/s23020715

**Published:** 2023-01-08

**Authors:** Min Li, Zheng Liu, Yu Xia, Mingyang He, Kangwen Yang, Shuai Yuan, Ming Yan, Kun Huang, Heping Zeng

**Affiliations:** 1 Shanghai Key Laboratory of Modern Optical System, Engineering Research Center of Optical Instrument and System, Ministry of Education, School of Optical-Electrical and Computer Engineering, University of Shanghai for Science and Technology, Shanghai 200093, China; 2 Jinan Institute of Quantum Technology, Jinan 250101, China; 3 State Key Laboratory of Precision Spectroscopy, East China Normal University, Shanghai 200062, China; 4 Chongqing Key Laboratory of Precision Optics, Chongqing Institute of East China Normal University, Chongqing 401121, China

**Keywords:** THz ranging, adaptive, dual-comb configurations

## Abstract

We propose and implement a terahertz time-of-flight ranging system based on adaptive clock asynchronous optical sampling, where the timing jitter is corrected in real time to recover the depth information in the acquired interferograms after compensating for laser instabilities using electronic signal processing. Consequently, the involved measurement uncertainties caused by the timing jitter during the terahertz sampling process and the noise intensity of the terahertz electric field have been reduced by the utilization of the adaptive clock. The achieved uncertainty range is about 2.5 μm at a 5 cm distance after averaging the acquisition time of 1876 ms 5000 times, showing a significant improvement compared with the asynchronous optical sampling using a constant clock. The implemented terahertz ranging system only uses free-running mode-locked lasers without any phase-locked electronics, and this favors simple and robust operations for subsequent applications that extend beyond the laboratory conditions.

## 1. Introduction

Light detection and ranging (LiDAR) has been successfully implemented in research on the microwave and infrared bands. The use of LiDAR plays a vital role in many applications, including inter-satellite ranging, the precision machining of industrial production, and the inspection of aerospace equipment [1,2,3,4]. Recently, an increased amount of attention has been paid to extending LiDAR’s operation into the terahertz (THz) wavelength region due to the pertinent advantages it could bring to taking nondestructive and internal measurements [5,6,7]. In combination with the features of nonionizing photon energies and the transparency of most of the nonconductive materials [8], THz ranging can empower three-dimensional tomography for applications such as system inspections and medical imaging [9,10]. However, the THz regime is plagued by a lack of sensitive detectors with low timing jitters, which imposes a great challenge in achieving high-precision distance measurements [11].

It has been proven in the infrared band that the time-of-flight (TOF) ranging of the elapsed time between optical pulses could result in large ambiguities, but high precision can be achieved with the help of dual-comb configurations [12,13,14,15]. The involved technique of asynchronous optical sampling (ASOPS) has also been adopted in THz generation and detection, which is referred to as the THz dual-comb system (THz-DCS) due to the stability of the carrier-envelope phase [16,17,18,19,20]. Recently, the THz-DCS has been adopted in THz TOF ranging research to measure the distance of stationary objects, exhibiting an accuracy of −551 μm and a resolution of 113 μm [21]. However, the accuracy and resolution are limited by the timing jitter during optical sampling due to the inevitable distortion and drifting of the THz pulse in the time domain [22,23,24]. To date, various schemes have been proposed to improve the accuracy in both the infrared and THz dual-comb systems [24,25,26,27]. For the THz quantum cascade laser (QCL) dual-comb system, actively stabilized, self-referenced, and adaptive approaches have been developed to generate a more stable comb in the time and frequency domains by canceling the phase noise and frequency drifting [28,29,30]. In contrast to the THz QCL dual-comb systems, THz generation based on optical pulse pumping offers a more straightforward method to produce a phase-stable THz comb, thus relaxing the stringent requirement of using high-accuracy feedback to maintain mutual coherence. In exchange, the residual timing jitter and intensity fluctuation slightly affect the sampling accuracy of the THz pulse. In this context, there is an pressing demand to develop a practical approach to improve the timing accuracy in THz TOF ranging to promote its broader application.

Here, an adaptive clock signal is prepared to achieve real-time compensation for the timing jitter in a THz TOF ranging system based on asynchronous optical sampling, consequently improving the measurement accuracy. In this scenario, adaptive clock signals originate from mixing radio-frequency beat notes between two single-frequency continuous waves and free-running mode-locked femtosecond pulses around the 1.5 μm wavelength. Its performance is similar to a system with a fully stabilized repetition rate. Furthermore, the intensity noise in the THz electric field is effectively suppressed through the benefits of multiple averaging during timing correction, which has also been proven to improve the measurement accuracy. The maximum residual of the measurement within the interval distance of 5 cm is reduced to 3.5 μm, and the uncertainty reaches 2.5 μm after averaging the acquisition time of 1876 ms 5000 times, indicating the feasibility of using the adaptive clock scheme for time correction.

## 2. Experimental Setup

**Figure 1:**
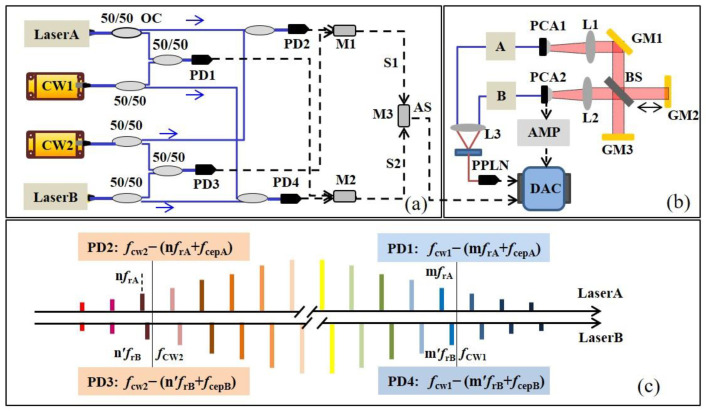
Schematic diagram of THz TOF ranging system with adaptive clock asynchronous sampling. (**a**) The diagram of adaptive clock generation. Laser A and Laser B are femtosecond pulse lasers with central wavelength 1550 nm, pulse duration 100 fs, and repetition rates of 66.140170 MHz and 66.139997 MHz, respectively; CW1 and CW2 are continuous wave lasers with wavelengths 1550 nm and 1564 nm, respectively; PD1,2,3,4, Photodetector; M, mixer; S, beat note signal; AS, adaptive clock signal. (**b**) The optical structure of THz TOF ranging system; A and B mean the femtosecond pulses from Laser A and Laser B, respectively; PCA, Photoconductive antenna; L, TPX lens; BS, THz beam splitter; GM, Gold mirror; PPLN, Periodically Poled Lithium Niobate crystal; AMP, transimpedance amplifiers; DAC, data acquisition card. (**c**) The beat note signals detected by PD1,2,3,4; m*f*_rA_ and n*f*_rA_ are frequencies of the “m”th and “n”th combs in Laser A, respectively; m′*f*_rB_ and n′*f*_rB_ are the frequencies of the “m′”th and “n′”th combs in Laser B, respectively; *f*_CW1_ and *f*_CW2_ are the frequencies of CW1 and CW2, respectively; *f*_cep_ means the carrier–envelope phase of Laser A and Laser B.

The diagram of the proposed THz TOF ranging scheme with adaptive clock asynchronous sampling is shown in Figure 1; the figure shows the adaptive clock generation process in Figure 1a and the optical part in Figure 1b. As shown in Figure 1a, two continuous lasers with different wavelengths act as an optical bridge to obtain relative beat notes between optical pulses. Light from narrow linewidths CW1 (1550 nm, 30 mW, linewidth < 5 kHz) and CW2 (1564 nm, 30 mW, linewidth < 5 kHz), as well as Lasers A and B, are split in multiple ways and combined to beat against each other; the beat notes from Laser A and B are achieved by an electronic process. As shown in Figure 1c, the beat notes between two continuous lasers and two pulse lasers in the experiment are recorded as *f*_PD1_, *f*_PD2_, *f*_PD3_, and *f*_PD4_, successively. *f*_PD1_ and *f*_PD4_ denote the beat notes between the lasers from CW1 and Laser A or Laser B, respectively, while *f*_PD2_ and *f*_PD3_ refer to the beat notes between the lasers from CW2 and Laser A or Laser B, respectively. The beat notes detected by PD can be expressed as:

*f*_PD1_ = *f*_CW1_ − (m*f*_rA_ + *f*_cepA_) (1)

*f*_PD2_ = *f*_CW2_ − (n*f*_rA_ + *f*_cepA_) (2)

*f*_PD3_ = *f*_CW2_ − (n′*f*_rB_ + *f*_cepB_) (3)

*f*_PD4_ = *f*_CW1_ − (m′*f*_rB_ + *f*_cepB_) (4)

where *f*_r_ is the repetition rate and *f*_cep_ is the carrier–envelope phase of Laser A and B. In order to avoid confusion due to mixing frequencies, the difference between the serial numbers of the beating teeth of the two combs is equal (m − n = m′ − n′) [31,32]. Afterwards, the effect of CW1 can be eliminated by mixing *f*_PD1_ and *f*_PD4_ as follows:

*f*_S2_ = *f*_PD4_ − *f*_PD1_ = m*f*_rA_ − m′*f*_rB_ + Δ*f*_cep_ (5)

while the effect of CW2 can be eliminated by mixing *f*_PD2_ and *f*_PD3_ as follows:

*f*_S1_ = *f*_PD3_ − *f*_PD2_ = n*f*_rA_ − n′*f*_rB_ + Δ*f*_cep_ (6)

In consideration of the phase stability of the THz pulse, the phase fluctuation of the femtosecond pulses should be further eliminated by mixing S1 and S2, only conserving the difference in the repetition rate. The generated adaptive clock signal AS could be expressed as:

*f*_AS_ = *f*_S2_ − *f*_S1_ = (m − n) (*f*_rA_ − *f*_rB_) = kΔ*f*_r_ (7)

where Δ*f*_r_ is the difference in the repetition rate between *f*_rA_ and *f*_rB_, and the AS signal consists of integer multiples of Δ*f*_r_, therefore containing their noises (represented as the jitter in the time domain). This AS signal fluctuates, synchronized with the timing jitter of the THz pulse sampled in the asynchronous sampling system in time. It acts as an adaptive clock, taking the place of the constant clock to compensate for the residual timing jitter, thus, a THz pulse corrected in real time will be detected.

The optical path is depicted in Figure 1b. Here, a THz pulse radiates from a fiber-coupled, strip-line-shaped LT-InGaAs/InAlAs photoconductive antenna (PCA1, TERA 15-TX-FC, MenloSystems, bias voltage = 95 V, optical power of 22 mW), which is pumped by femtosecond pulses from Laser A. The parallel THz beam after L1 and gold mirror 1 (GM1) is divided into two pulses by a THz beam splitter (BS). The THz pulse reflected back from GM3 acts as a reference pulse, and the pulse reflected by GM2 is placed on a stepper motor (UTS50CC, Newport) to obtain dynamic measurement, serving as the measurement pulse. The reference and measurement pulses recombine at the BS and are refocused on another fiber-coupled, dipole-shaped LT-InGaAs/InAlAs photoconductive antenna (PCA2, TERA 15-RX-FC, MenloSystems, optical power of 25 mW), which is pumped by Laser B. The repetition rate of Laser A is *f*_rA_ (66.140170 MHz), and the repetition rate of Laser B acting as the detected light is *f*_rB_ (*f*_rB_ = 66.139997 MHz, *f*_rA_ = *f*_rB_ + Δ*f*_r_, Δ*f*_r_ = 173 Hz). The weak current from PCA2 is amplified by a transimpedance preamplifier (AMP, FEMTO, HCA-10-100K, noise current = 1.1 pA, bandwidth = 10 MHz, gain = 1 × 10^5^ V/A), and then, it is simultaneously connected to the data acquisition card (DAC, Alazar, ATS9462, sample frequency = 80 Msp) together with the adaptive clock signal. Portions of the coherent synchronous lights from the two pulse lasers are used to generate a sum-frequency generation cross-correlator (SFG-X). During the acquisition of the THz pluses, the SFG acts as a trigger signal to provide a time origin signal to ensure the accuracy of the real-time THz pluses. The reference pulse and the measurement pulse are obtained in every 1/Δ*f*_r_ measurement period. The final ranging result can be calculated as:

D_TOF_ = (*c*/2*n*_g_) × Δ*t* × (*f*_rA_/Δ*f*_r_) (8)

where *c* is the group velocity of the light pulse, *n*_g_ = 1.000264 is the air group refractive index, Δ*t* is the time interval between the reference pulse and measurement pulse, and *f*_rA_/Δ*f*_r_ is the time amplification factor, while the THz region diminishes to the radio frequency (RF) region.

Furthermore, we designed a real-time feedback loop to avoid exceeding the bandwidth of the filters and used a piezo-ceramic transducer (PZT) and stepper motor (SM) to actively rectify the slow drifting of pulse lasers in order to ensure that the relevant electronics operate within a workable bandwidth. Specifically, CW 2 was set to a fixed frequency by utilizing a constant voltage and a thermoelectric cooler (TEC). In addition, the beat notes *f*_PD2_ and *f*_PD3_ were loosely stabilized at the center frequency of the band filter by the PZT and SM, while they were drifting out. Meanwhile, *f*_PD1_ could also be stabilized within the filtering window through the control of CW 1. In this way, the frequencies of all four beat notes could be stabilized within the filtering window. Therefore, the adaptive clock can be extracted stably and reliably.

## 3. Results and Discussion

As noted above, the relative uncertainty (*U*/D_TOF_) of the TOF-based ranging results are expressed as follows [33,34]:

(9) $$\frac{U}{D_{\mathrm{TOF}}}\approx \sqrt{{\left[\frac{u_{v_{g}}}{v_{g}}\right]}^{2}+{\left[\frac{u_{\Delta t}}{\Delta t}\right]}^{2}+{\left[\frac{u_{\Delta f_{r}}}{\Delta f_{r}}\right]}^{2}+{\left[\frac{u_{\Delta f_{rA}}}{f_{rA}}\right]}^{2}}$$

where *u_v_*_g_, *u*_Δ*t*_, *u*_Δ*f*r_, and *u_f_*_rA_ represent the uncertainties of *v*_g_, Δ*t*, Δ*f*_r_, and *f*_rA_, respectively. Obviously, the relative uncertainty originating from the pulse velocity uncertainty caused by the environmental disturbance could be ignored (10^−7^). The impact from the uncertainty of the repetition rate depends on the accuracy of the radio frequency reference (10^−11^), which could be also ignored. Although it is different from the above two parameters, the uncertainty of Δ*t* is affected by the timing jitter and intensity fluctuations of the THz pulse. The drifting of the repetition rate offset Δ*f*_r_ in the asynchronous system also partly impacts the relative uncertainty.

**Figure 2:**
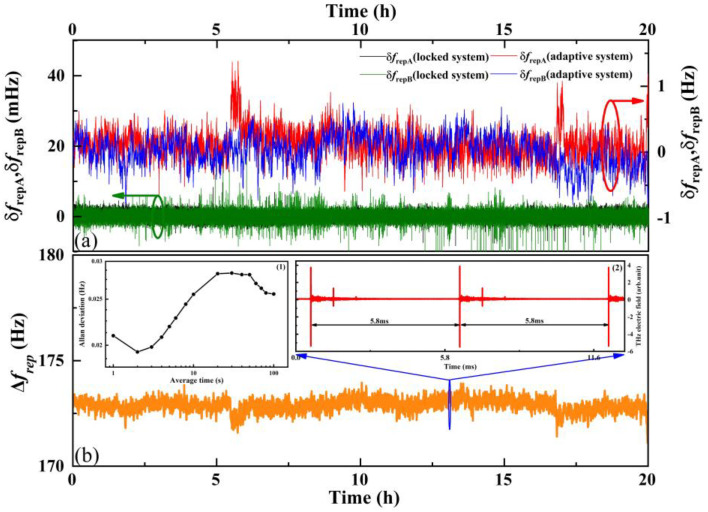
Fluctuations of *f*_rA_, *f*_rB_, and Δ*f*_r_. (**a**) Frequency instability of *f*_rA_ and *f*_rB_ in two different systems: locked system means the repetition rate of lasers are locked, and adaptive system means lasers are in free-running, mode-locked status. (**b**) Instability of repetition rate difference Δ*f*_r_ in adaptive system. The inset (1) shows the calculated Allan deviation of Δ*f*_r_ in adaptive system ranging from 1 s to 100 s, and (2) shows the sampling THz electric field during this process.

Firstly, to demonstrate the time correction effect of the adaptive clock, two THz TOF ranging systems excited by different laser systems (here, the system using locked repetition rate lasers is named the locked system, and the system based on adaptive clock asynchronous sampling is named the adaptive system) are utilized to analyze the effect on measurement accuracy. We used two synchronized counters (Tektronix FCA3000, 1-s gate) to record the repetition rate of the pulse lasers with the measurement accuracy in mHz. As illustrated in Figure 2a, the instability of the repetition rate is measured continuously for 20 h to reflect the performance of the lasers in both of the systems. The black and green curves in Figure 2a show that the peak-to-peak repetition rates fluctuate in the locked system (locked to 75.420060 and 75.420050 MHz, respectively), and they are stable at ±10 mHz, with a standard deviation of 1.31 mHz. As they are significantly different from the locked system, the peak-to-peak repetition rates of the adaptive system (maintained around 66.140170 and 66.139997 MHz, respectively) fluctuate within ±1 Hz, with a standard deviation of 207.35 mHz, as shown by the blue and red curves in Figure 2a. The relative uncertainties caused by the jitter of *f*_rA_ from the locked and adaptive systems are of the magnitude of 10^−11^ and 10^−8^, respectively. Secondly, we analyzed the relative uncertainty caused by the instability of the repetition rate difference. As shown in Figure 2b, the Δ*f*_r_ of the adaptive system fluctuates around 173 Hz, with a standard deviation of 0.27532 Hz, bringing about a relative uncertainty on the magnitude of 10^−3^, which is higher than that of the locked system (10^−5^), and this unavoidably causes a worse performance in the ranging process.

**Figure 3:**
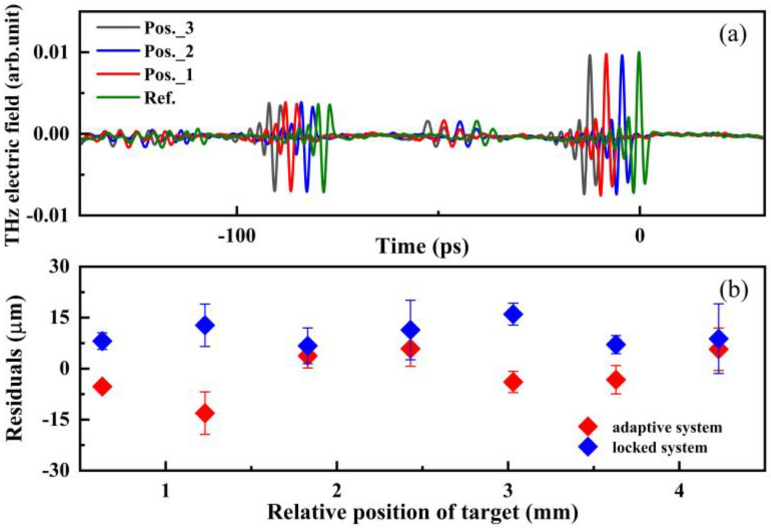
(**a**) THz signals sampled by THz TOF ranging with adaptive clock asynchronous sampling. Green curve represents the reference signal; black, blue, and red curves represent the measurement signals at different positions. (**b**) The measurement results of adaptive system (red) and locked system (blue).

The measurement accuracy of the THz TOF ranging system was recorded accordingly. The step size of the step motor is set at 600 μm (motor standard accuracy ± 1.1 μm), and 1000 groups are sampled and averaged. Figure 3a shows the results measured by the adaptive system; the reference signal is indicated by the green line, and the moving measurement signals are represented by the curved lines in black, blue, and red, respectively. The measured distance between pos_1 and pos_2 is 593 μm, and that one between pos_2 and pos_3 is 609 μm. Then, seven positions (equally separated by 0.6 mm) are measured to compare the measurement accuracy by the THz TOF ranging based on adaptive and repetition-rate locked techniques, respectively. The deviation from the true value of the standard movement of the motor is plotted in Figure 3b. The statistical uncertainty for each point (10 individual measurements for each position) is evaluated, plotted as the error bar, and depicted in Figure 3b. The uncertainty of the blue error bar distribution is about 3.4 μm, and it is also about 3.4 μm for the red one. As mentioned above, the results of the TOF ranging are mainly influenced by the uncertainty of Δ*t* and Δ*f*_r_. In fact, the fluctuation of Δ*f*_r_ and Δ*t* in the adaptive system is about 100 times larger than that in the repetition-rate-locked system. Still, the uncertainty maintains similar characteristics, verifying the validated compensation of the adaptive clock in the fluctuation of Δ*f*_r_ and Δ*t*.

**Figure 4:**
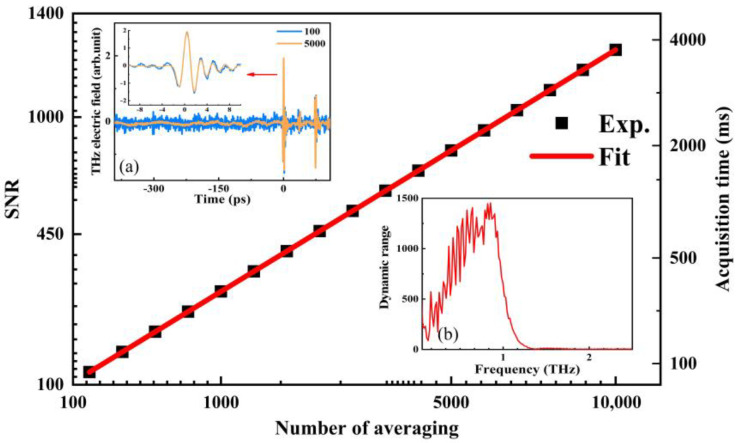
The SNR of THz time domain electric field increases as a square root of the averaging numbers and acquisition time. The inset (**a**) shows the sampling THz electric field at different averaging times, and (**b**) it shows the dynamic range at the acquisition time of 1876 ms.

The intensity noise in the THz electric field is another important element impacting the uncertainty of Δ*t* in addition to the timing jitter in TOF ranging. This uncertainty can be reduced by performing averaging through multiple measurements. However, the accumulation of residual timing jitter in a locked system reduces the coherence of the system and makes it impossible to average through multiple measurements. The compensation of the timing jitter in the adaptive system makes such averaging feasible. In addition, the capability of the multiple coherent averaging in the time domain enhances the time domain signal-to-noise ratio (SNR) of the sampled THz pulse. In the adaptive system, we acquired multiple signals at a fixed location and plotted the SNR versus the average time, as shown in Figure 4. Here, we define the SNR as the peak-to-peak value of the signal divided by the standard deviation (SD) of the noise away from the signal. In the diagram, the SNR is proportional to the square root of the averaging numbers and greatly improves with the increase in number of averaging instances. In the illustration, the THz electric field is averaged 100 and 5000 times, and the noise decreases gradually with the increase in the number of averaging instances, showing the effective suppression of noise intensity.

**Figure 5:**
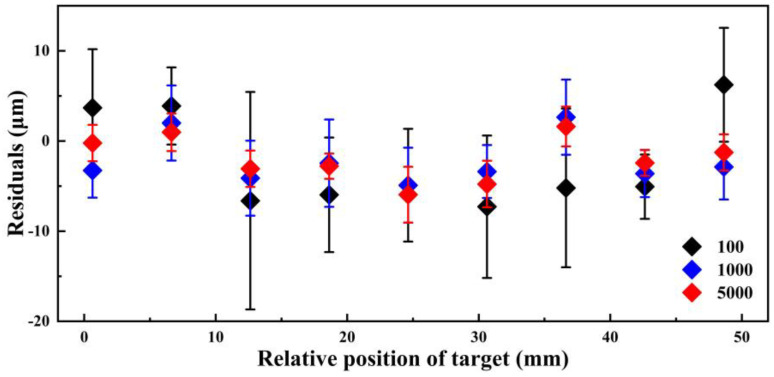
Distance measurement results of THz TOF ranging system based on adaptive clock asynchronous sampling with averaging counts of 100 (black), 1000 (blue), and 5000 (red).

To confirm the influence of the SNR on the uncertainty of THz TOF ranging, we measure the THz signals, each one being averaged 100, 1000, and 5000 times in 10 positions (equally separated by 6 mm). The deviation from the true value of the standard movement of the motor is plotted in Figure 5, and the final range error uncertainties for the averaging times of 100, 1000, and 5000 are about 5.3, 2.7, and 2.5 μm, respectively. It is also confirmed that the fluctuations in Δ*t* are compensated for as the number of averages increases, thus reducing the range error and uncertainty.

## 4. Conclusions

In summary, we have experimentally verified the real-time correction feasibility of an adaptive clock signal in THz TOF ranging with asynchronous optical sampling. The sampling uncertainty caused by the timing jitter and the noise intensity of the THz electric field could be effectively reduced by the real-time compensation of the time interval and random-noise reduction with time averaging. Furthermore, the multiple averaging ability of the adaptive system may greatly improve the detection sensitivity and increase the operation range, thus favoring the implementation of THz laser ranging. Moreover, considering its good penetrability in various dielectric materials and nonpolar substances, the presented THz TOF ranging system would provide a powerful instrument for the nondestructive observation and contactless measurement of opaque objects, bringing new opportunities in applications in the field.

## Data Availability

Not applicable.
